# Regressed coronary ostial stenosis in a young female with Takayasu arteritis: a case report

**DOI:** 10.1186/s12872-019-1066-7

**Published:** 2019-04-02

**Authors:** Tetsuro Yokokawa, Hiroyuki Kunii, Takashi Kaneshiro, Shohei Ichimura, Akiomi Yoshihisa, Makiko Yashiro Furuya, Tomoyuki Asano, Kazuhiko Nakazato, Takafumi Ishida, Kiyoshi Migita, Yasuchika Takeishi

**Affiliations:** 1 Department of Cardiovascular Medicine, Fukushima Medical University, 1 Hikarigaoka, Fukushima, Fukushima 960-1295 Japan; 2 Department of Pulmonary Hypertension, Fukushima Medical University, Fukushima, Japan; 3 Department of Advanced Cardiac Therapeutics, Fukushima Medical University, Fukushima, Japan; 4 Department of Rheumatology, Fukushima Medical University, Fukushima, Japan

**Keywords:** Takayasu arteritis, Coronary ostial stenosis, Regression, Tocilizumab, ^18^F-fluorodeoxyglucose positron emission tomography/computed tomography, Case report

## Abstract

**Background:**

Takayasu arteritis is a rare systemic vasculitis, which affects the aorta and its major branches, especially in young females. Diagnosis and treatment for Takayasu arteritis with coronary stenosis are important to prevent fatal complications. Immunosuppressive treatment such as corticosteroid is a common treatment for this condition. However, the effects of immunosuppressive treatment on inflammatory coronary stenosis caused by Takayasu arteritis remains unknown.

**Case presentation:**

An 18-year-old female had chest oppression on effort and was referred to our hospital due to ST-segment depression in I, aV_L_, and V_2–4_ on electrocardiogram. Coronary angiography showed severe stenosis in the ostium of both the left main trunk and the right coronary artery. ^18^F-fluorodeoxyglucose (FDG) positron emission tomography/computed tomography showed isolated inflammation of the aortic root. She was diagnosed with Takayasu arteritis and treated with combined immunosuppressive treatment with corticosteroid and tocilizumab, which decreased the FDG uptake in the aortic root. Four months after initiation of the immunosuppressive treatment, coronary angiography showed regression of the coronary ostial stenosis. Coronary artery bypass surgery was considered, but the patient rejected invasive revascularization for coronary artery disease. She did not have chest oppression or ST-segment depression after the immunosuppressive treatment. She had no cardiac events for 6 months after discharge.

**Conclusions:**

We described regressed coronary ostial stenosis in a young female patient with Takayasu arteritis. Immunosuppressive treatment might have a favorable effect on coronary ostial stenosis in Takayasu arteritis.

## Background

Takayasu arteritis is a rare disease that typically occurs in young females, affecting the aortic arch and proximal branches of the aorta, as well as pulmonary arteries. The incidence rate of coronary artery disease in Takayasu arteritis is 10 to 45% in autopsy cases [1, 2]. Coronary artery stenosis is due to the extension of the inflammatory process and intimal proliferation in the ascending aorta. Seventy-three percent of occlusive coronary artery diseases are localized around the coronary ostium, and coronary artery stenosis is sometimes fatal [1, 2]. ^18^F-fluorodeoxyglucose (FDG) positron emission tomography (PET)/computed tomography (CT) is widely used for malignant and inflammatory diseases, by which we can detect active vasculitis and monitor disease activity [3]. ^18^F-FDG PET/CT is also useful for evaluating inflammatory coronary lesions.

For the treatment of Takayasu arteritis, prednisolone has been used in 79 to 94% of patients to control disease activity [4, 5]. Among biologic drugs, tocilizumab, an anti-interleukin-6 receptor antibody, has been reported to be effective for the treatment of refractory Takayasu arteritis [6]. The effectiveness of the immunosuppressive treatment, including prednisolone and tocilizumab, is unknown for inflammatory coronary arterial lesions. One anecdotal report described the regression of coronary artery stenosis evaluated by CT after immunosuppressive treatment [7]. Among invasive revascularization techniques, percutaneous coronary intervention with coronary stent has a high risk of restenosis [8]. For patients with severe coronary artery stenosis, coronary artery bypass surgery is considered after suppression of disease activity [9, 10]. There is no established treatment for coronary arterial lesions for favorable long-term prognosis in young patients with Takayasu arteritis. We present a rare case of a young female with Takayasu arteritis who had regressed coronary ostial stenosis after immunosuppressive treatment and was evaluated by repeated coronary angiography and ^18^F-FDG PET/CT.

### Case presentation

**Fig. 1:**
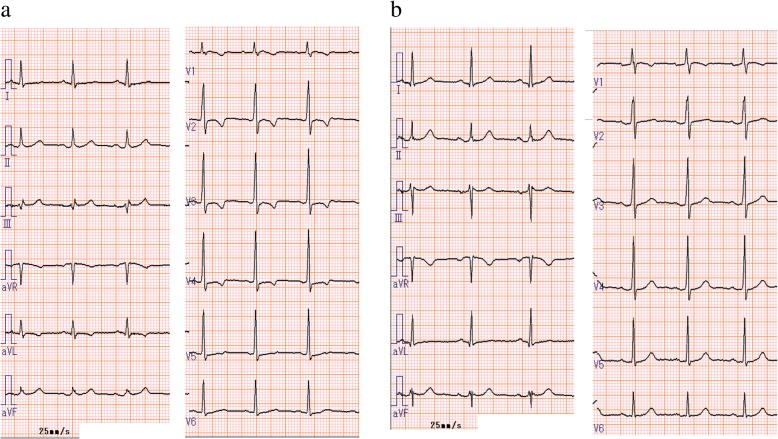
Electrocardiogram. **a**. Electrocardiogram showed ST-segment depression in leads I, aV_L_, and V_2–4_. **b**. Electrocardiogram showed no ST-segment depression after the treatment

**Fig. 2:**
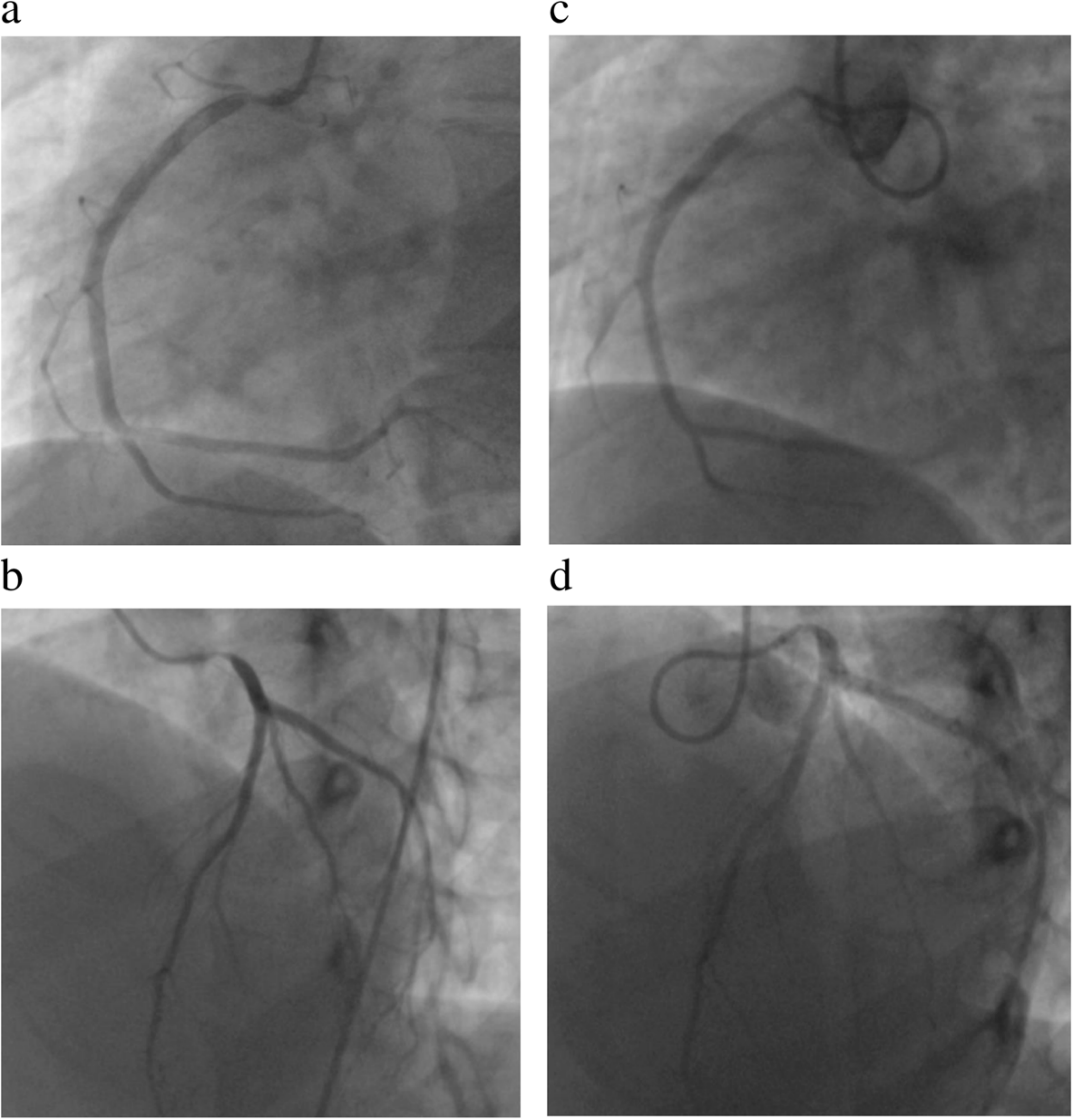
Coronary angiography. **a**, **b**. Initial coronary angiography showed severe stenosis in the ostium of both the left main trunk and the right coronary artery. **c**, **d**. Four months after immunosuppressive treatment, coronary angiography showed significant regression of the right coronary ostial stenosis, but limited regression of the left coronary ostial stenosis

**Fig. 3:**
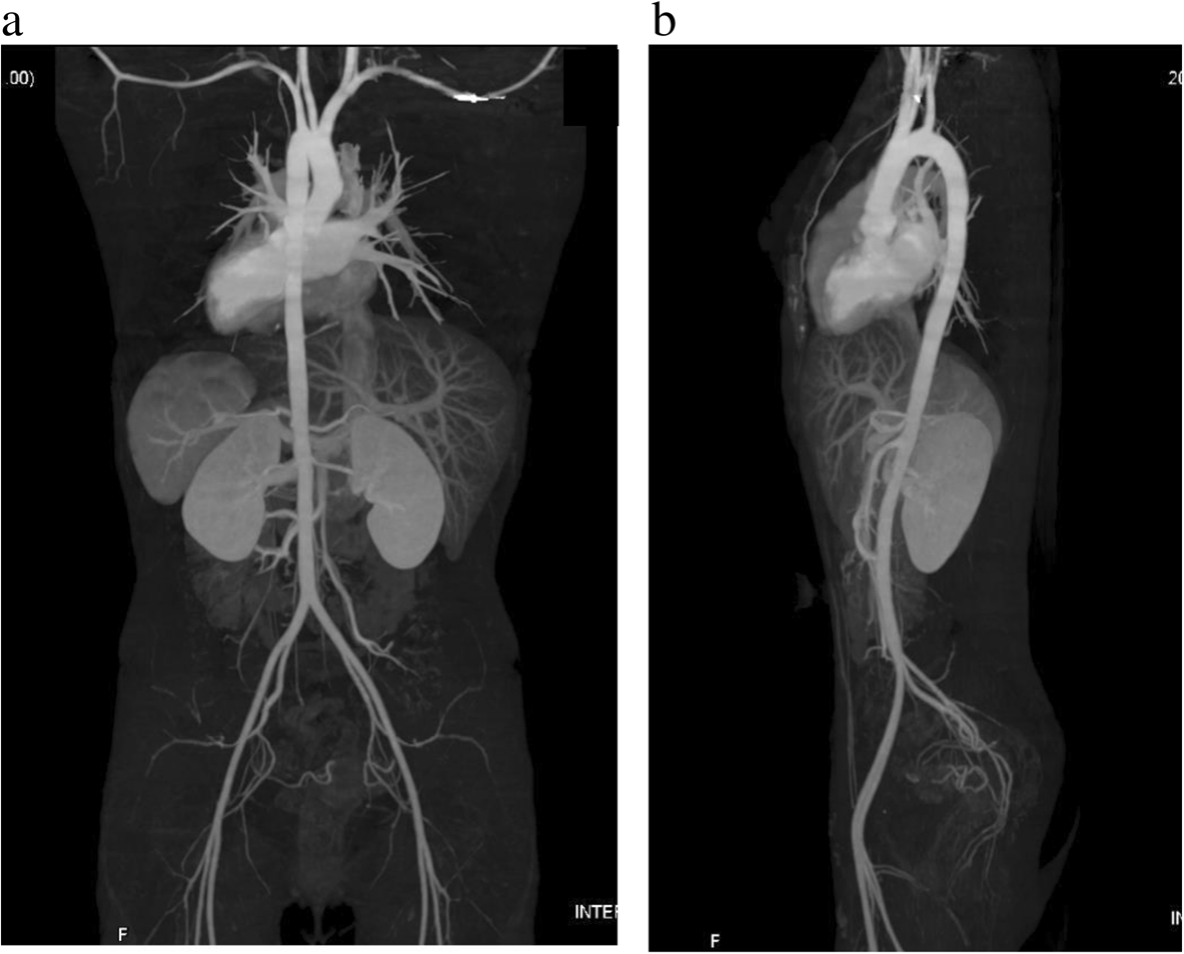
Contrast-enhanced computed tomography. **a**, **b**. No significant aortic lesion was detected by contrast-enhanced computed tomography

**Fig. 4:**
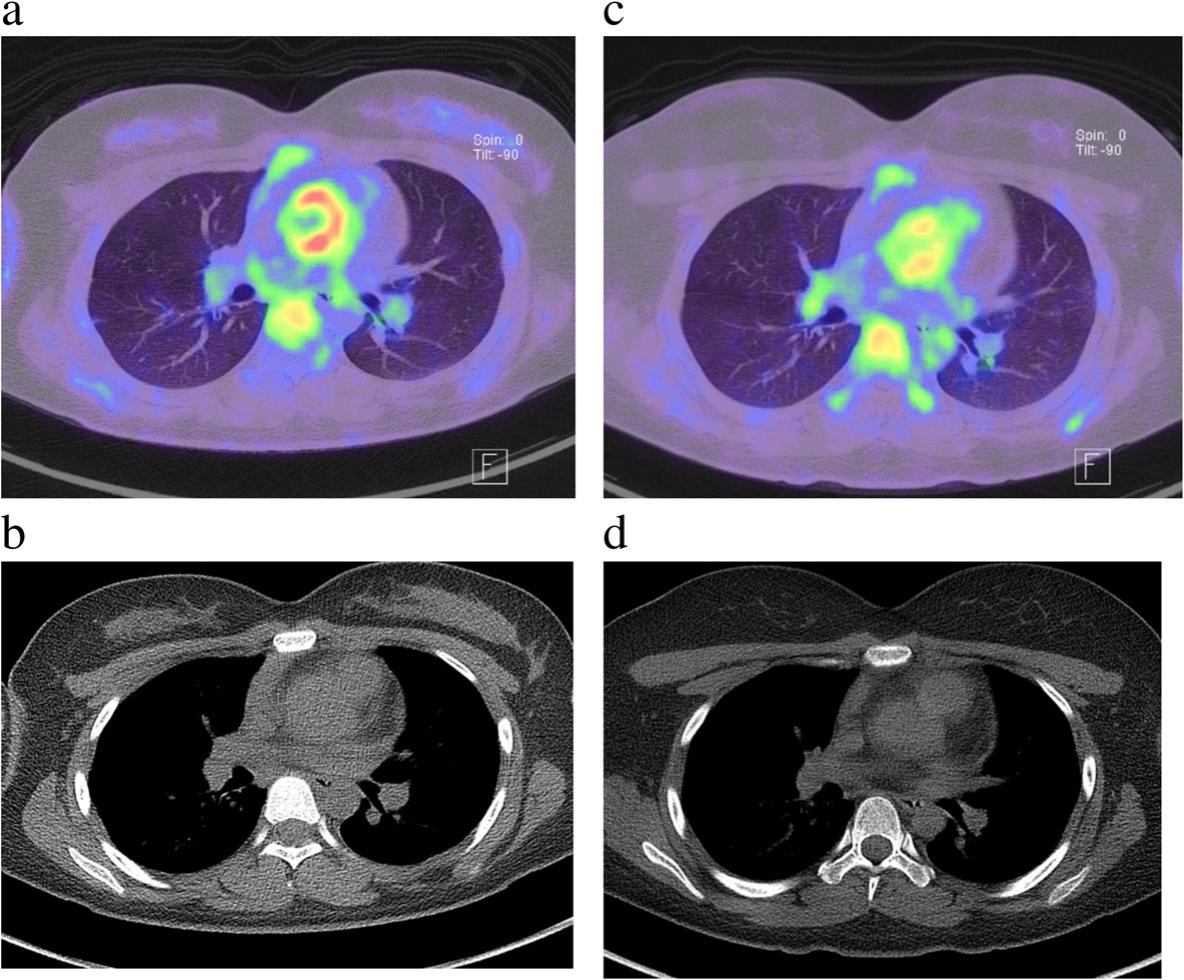
^18^F-fluorodeoxyglucose (FDG) positron emission tomography (PET)/computed tomography (CT). **a**, **b**. ^18^F-FDG PET/CT showed isolated inflammation of the aortic root on admission. The maximum standardized uptake value (max SUV) was 6.3. **c**, **d**. After immunosuppressive treatment, ^18^F-FDG PET/CT showed decreased ^8^F-FDG uptake in the aortic root. The max SUV was decreased to 4.3

**Fig 5:**
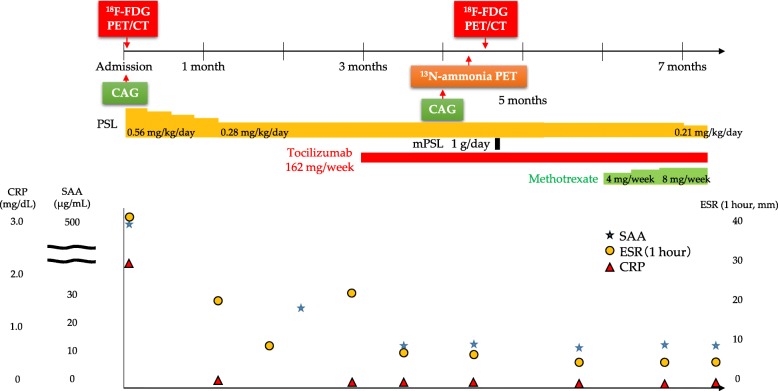
Clinical course. CAG, coronary angiography; CT, computed tomography; CRP, C-reactive protein; ESR, erythrocyte sedimentation rate; FDG, fluorodeoxyglucose; PET, positron emission tomography; PSL, prednisolone, SAA, serum amyloid A; mPSL, methylprednisolone

An 18-year-old female suffered from chest oppression on effort for a month. She visited a hospital, and her electrocardiogram showed ST-segment depression in leads I, aV_L_, and V_2–4_ (Fig. 1a). She was suspected of having angina pectoris, and was referred to our hospital. Her laboratory data on admission showed a normal range of creatinine kinase and troponin I as shown in Table 1. Echocardiography revealed normal left ventricular contraction and mild to moderate aortic regurgitation. Coronary angiography showed severe stenosis in the ostium of both the left main trunk and the right coronary artery (Fig. 2a, b). Quantitative coronary angiography analysis was performed with a computerized quantitative analysis system (QAngio XA version 7.3, Medis Medical Imaging System, Leiden, The Netherlands), using a contrast-filled catheter as a calibration source. The percentage of the diameter of the most severe stenosis compared with the reference diameter was defined as % diameter stenosis [11]. % diameter stenosis of the ostial stenosis was 95.0% in the left main trunk and 87.2% in the right coronary artery. Intra-coronary administration of isosorbide dinitrate did not dilate the coronary ostial stenosis, suggesting that the stenosis was an organic lesion. Optimal medical treatment, including beta-blocker, antiplatelet, and statin, was initiated for her coronary artery disease. Contrast-enhanced CT showed no specific abnormality of the aorta (Fig. 3a, b). She did not have renal artery stenosis or hypertension. Her right and left ankle-brachial indices were 1.08, and 1.03, respectively. She was examined by an ophthalmologist and did not have vision impairment. Magnetic resonance angiography revealed no significant stenosis of her carotid artery. The patient had no coronary risk factors or signs of infectious disease and congenital heart disease. C-reactive protein, serum amyloid A, and erythrocyte sedimentation rate (1 h) on admission were 2.13 mg/dL (normal range: 0.00–0.30 mg/dL), 479 μg/mL (normal range: 0.0–8.0 μg/mL), and 40 mm (normal range: 3–15 mm), respectively, suggesting systemic inflammation. ^18^F-FDG PET/CT showed isolated inflammation of the aortic root (Fig. 4a, b). She was therefore diagnosed with Takayasu arteritis, and oral administration of prednisolone was started from 0.56 mg/kg/day. Tocilizumab was added 3 months after the initiation of prednisolone (Fig. 5). Follow-up coronary angiography showed regression of the ostial stenosis 4 months after the initiation of prednisolone, and % diameter stenosis was 86.7% in the left main trunk and 72.6% in the right coronary artery (Fig. 2c, d). Intravascular ultrasound or optical coherence tomography was not performed for the ostial lesions. Her C-reactive protein and erythrocyte sedimentation rate (1 h) were decreased to 0.03 mg/dL and 5 mm, respectively. The second ^18^F-FDG PET/CT showed decreased ^18^F-FDG uptake in the aortic root, but still showed inflammation (Fig. 4c, d). Her serum amyloid A of 13.8 μg/mL was not normalized, therefore we decided to control the disease activity by combined immunosuppressive treatment including steroid pulse therapy (methylprednisolone 1 g/day for 3 days). Oral methotrexate ranging from 4 to 8 mg/week was administered to decrease corticosteroid dose (Fig. 5). The levels of serum amyloid A were not significantly increased and the dose of prednisolone was decreased from 0.28 to 0.21 mg/kg/day after administration of methotrexate.

**Table 1:** Laboratory findings on admission

Parameters	Value
Blood count	
White cell count	10,100 /μL
Red blood cell	451 × 10^4^ /μL
Hemoglobin	12.0 g/dL
Hematocrit	37.1%
Platelet count	37.4 × 10^4^ /μL
Biochemistry	
AST	16 IU/L
ALT	13 IU/L
LDH	248 IU/L
ALP	275 IU/L
Total bilirubin	0.7 mg/dL
Direct bilirubin	< 0.1 mg/dL
BUN	9 mg/dL
Creatinine	0.71 mg/dL
eGFR	91 mL/min/1.73 m^2^
Sodium	141 mEq/L
Potassium	4.4 mEq/L
Chlorine	103 mEq/L
Total protein	7.4 g/dL
Albumin	3.4 g/dL
Uric acid	5.0 mg/dL
Creatine kinase	54 IU/L
CK-MB	< 0.5 IU/L
Troponin I	0.035 ng/mL
BNP	10.2 pg/mL
Triglyceride	143 mg/dL
HDL-C	39 mg/dL
LDL-C	111 mg/dL
CRP	2.13 mg/dL
FT3	2.81 pg/mL
FT4	1.32 ng/mL
TSH	3.510 U/mL
Glucose	109 mg/dL
HbA1c (NGSP)	5.5%
ESR (1 h)	40 mm
SAA	479 μg/mL

*AST* aspartate transaminase, *ALT* alanine aminotransferase, *ALP* alkaline phosphatase, *BNP* B-type natriuretic peptide, *BUN* blood urea nitrogen, *CRP* C-reactive protein, *CK-MB* creatine kinase MB, *eGFR* estimate glomerular filtration rate, *ESR* erythrocyte sedimentation rate, *FT3* free triiodothyronine, *FT4* free thyroxine, *HbA1c* hemoglobin A1c, *HDL-C* high-density lipoprotein, *LDH* lactate dehydrogenase, *LDL-C* low-density lipoprotein, *SAA* serum amyloid A, *TSH* thyroid stimulating hormone, *NGSP* national glycohemoglobin standardization program

**Fig. 6:**
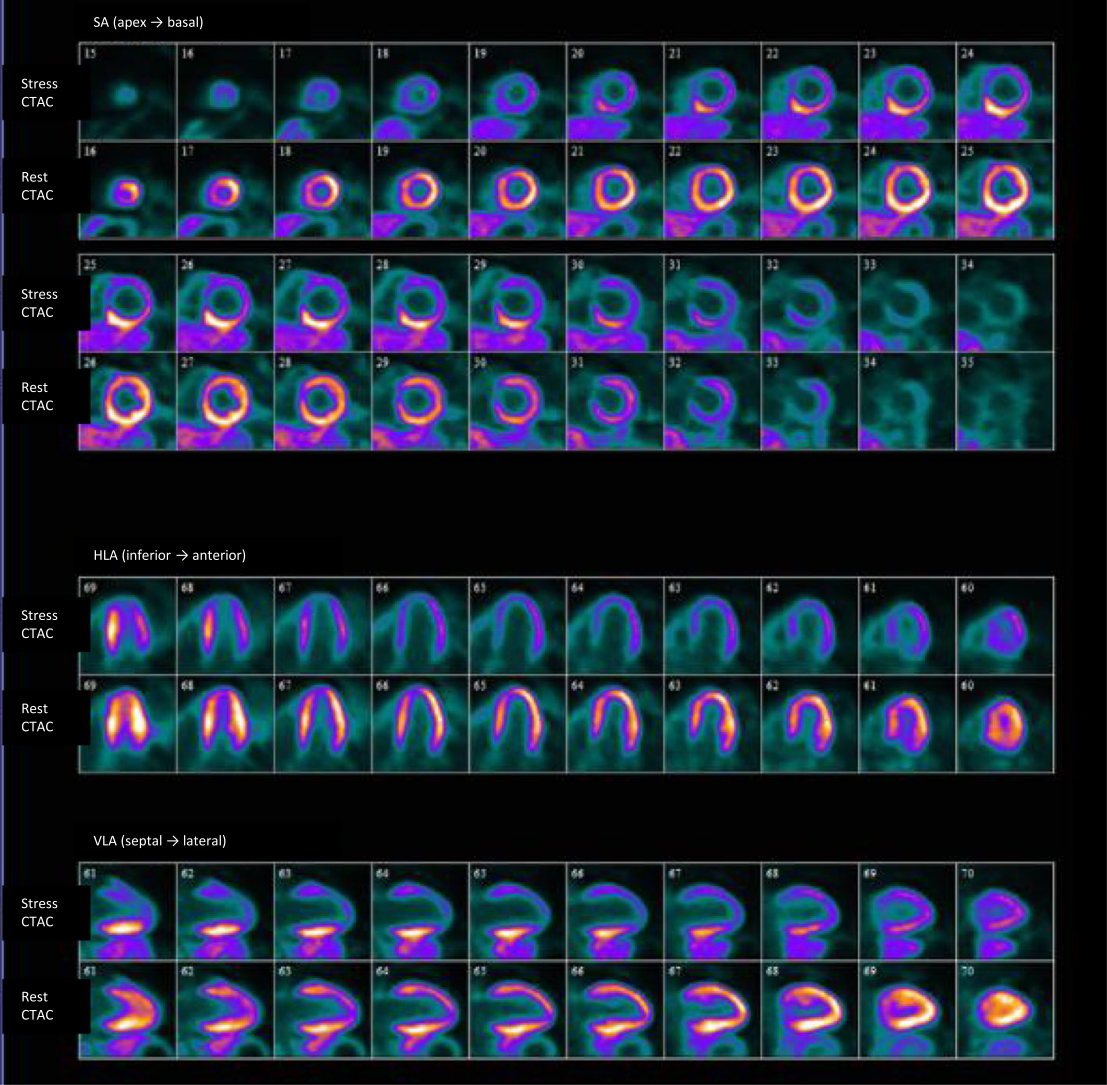
Myocardial perfusion imaging with ^13^N-ammonia positron emission tomography. Stress and rest ^13^N-ammonia perfusion imaging demonstrated significantly decreased uptake in the anterior and lateral lesions and slightly decreased uptake in the inferior lesion. CTAC, computed tomography-based attenuation correction; HLA, horizontal long axis; SA, short axis; VLA, vertical long axis

Myocardial perfusion imaging with ^13^N-ammonia PET detected myocardial ischemia (Fig. 6). Coronary artery bypass surgery was considered after the suppression of disease activity, but the patient and her family rejected invasive revascularization for coronary artery disease. After immunosuppressive treatment with prednisolone, tocilizumab and methotrexate, chest oppression and ST-segment depression was not observed (Fig. 1b). She was discharged after a 201-day hospitalization and administered 0.21 mg/kg/day of prednisolone, 8 mg/week of methotrexate, 162 mg/week of tocilizumab, 40 mg/day of isosorbide dinitrate, 15 mg/day of nicorandil, 100 mg/day of aspirin, 2.5 mg/day of rosuvastatin, and 10 mg/day of carvedilol. Electrocardiogram at rest revealed no significant ST-T change, and laboratory data showed no significant increase of C-reactive protein, erythrocyte sedimentation rate (1 h), or serum amyloid A in the ambulatory follow-up. She had no cardiac events for 6 months after discharge.

## Discussion and conclusions

In the present case, coronary ostial stenosis of Takayasu arteritis was regressed after immunosuppressive treatment in accordance with the improvement of the inflammation evaluated by repeated ^18^F-FDG PET/CT. This case suggests that inflammatory coronary stenosis might be reversible, and we could avoid early invasive revascularization for coronary artery disease in a case of active Takayasu arteritis. In our previous paper, we have focused only on the diagnosis using PET/Magnetic resonance imaging with ^18^F-FDG [12].

Because of the wide variation of clinical course, the diagnosis and management of Takayasu arteritis are still challenging [13, 14]. The effects of immunosuppressive treatment on inflammatory coronary stenosis caused by Takayasu arteritis remain unknown. Several previous case reports showed that arterial stenosis could be reversible in patients with Takayasu arteritis. Carotid artery stenosis has been reported to regress after corticosteroid therapy [15]. Immunosuppressive treatment improved stenotic lesions of the aorta in a case of a 3-month-old girl [16]. Regression of renal artery stenosis and abdominal aortic lesions has been reported after prednisolone treatment [17, 18]. In cases with coronary stenosis, only two anecdotal reports described regression in Takayasu arteritis. Isomatsu et al. described regression of coronary ostial stenosis after steroid therapy and coronary artery bypass surgery [19]. Mohan et al. reported regressed coronary ostial stenosis with images from cardiac-gated CT after immunosuppressive treatment [7]. To our knowledge, the present case report is the first to show regressed coronary ostial stenosis using repeated coronary angiography and ^18^F-FDG PET/CT.

In the present case, we performed early ^18^F-FDG PET/CT, and corticosteroid therapy was initiated immediately after the diagnosis of Takayasu arteritis. Early diagnosis and treatment might be effective to prevent the progression of active coronary artery disease in patients with Takayasu arteritis. There are several blood inflammatory biomarkers such as C-reactive protein and erythrocyte sedimentation rate. C-reactive protein is produced in the liver in response to interleukin-6, and thus C-reactive protein is not suitable to evaluate inflammatory activity after initiation of tocilizumab. However, serum amyloid A is suitable to evaluate disease activity under tocilizumab treatment [20, 21]. In the present case, serum amyloid A and ^18^F-FDG uptake indicated that inflammation remained in the patient. So, the treatment with methylprednisolone and methotrexate was started. The measurement of serum amyloid A was useful for managing Takayasu arteritis with coronary ostial stenosis under tocilizumab treatment.

Myocardial perfusion imaging detected myocardial ischemia caused by residual stenosis in our case. Follow-up angiography revealed more significant regression of the ostial lesion of the right coronary artery than that of the left coronary artery, and myocardial perfusion imaging showed only a slightly decreased uptake in the inferior wall. In the case of the left coronary artery, the ostial lesion showed limited regression at the follow-up angiography, and myocardial perfusion imaging revealed significant reversible uptake in the anterior and lateral wall.

In conclusion, we described regressed coronary ostial stenosis in a young female with Takayasu arteritis. Immunosuppressive treatment might have a favorable effect on coronary ostial stenosis in Takayasu arteritis.

## Abbreviations

CT: Computed tomography
FDG: Fluorodeoxyglucose
PET: Positron emission tomography
